# *Vepris amaniensis*: a morphological, biochemical, and molecular investigation of a species complex

**DOI:** 10.7717/peerj.17881

**Published:** 2024-09-25

**Authors:** Mary Ciambrone, Moses K. Langat, Martin Cheek

**Affiliations:** 1 Queen Mary University of London, London, United Kingdom; 2 Accelerated Taxonomy, Royal Botanic Gardens at Kew, London, United Kingdom; 3 Trait Diversity and Function, Royal Botanic Gardens Kew, London, United Kingdom

**Keywords:** Vepris, Sanger sequencing, Biochemistry, Taxonomy, *Vepris usambarensis*, Phylogeny, ITS, *trnL-F*

## Abstract

*Vepris* Comm. ex A. Juss. is a genus of 96 species extending from Africa to India that are distinct in their unarmed stems and their digitately (1-)3(-5) foliolate leaflets, and whose many secondary compounds earn them uses in traditional medicine. Mziray (1992) subsumed six related genera into *Vepris*, with *Vepris amaniensis* (Engl.) Mziray becoming somewhat of a dustpan for ambiguous specimens (Cheek & Luke, 2023). This study, using material from the Kew herbarium, sought to pull out novel species from those previously incorrectly filed as *Vepris amaniensis*, and here describes the new species *Vepris usambarensis* sp. nov. This species is morphologically distinct from *Vepris amaniensis* with its canaliculate to winged petioles, 0.5–2.3 cm long inflorescences, 1–3 foliolate leaflets, and hairs on inflorescences and stem apices. Phytochemical analysis attributed seven compounds to *Vepris usambarensis*: tecleanthine (**1**), evoxanthine (**2**), 6-methoxytecleanthine (**3**), tecleanone (**4**), 1-(3,4-methylenedioxyphenyl)-1,2,3-propanetriol (**5**), lupeol (**6**), and arborinine (**7**). This is a unique mixture of compounds for a species of *Vepris*, though all are known to occur in the genus, with the exception of 1-(3,4-methylenedioxyphenyl)-1,2,3-propanetriol (**5**) which was characterized from a species in the Asteraceae. An attempt at constructing a phylogeny for *Vepris* using the ITS and *trnL-F* regions was made, but these two regions could not be used to differentiate at species level and it is suggested that 353 sequencing is used for further research. Originally more than one new species was hypothesized to be within the study group; however, separating an additional species was unsupported by the data produced. Further phylogenetic analysis is recommended to fully elucidate species relationships and identify any cryptic species that may be present within *Vepris usambarensis*.

## Introduction

*Vepris* Comm. ex A. Juss. is a genus consisting of 96 species (Plants of the World Online, 2023) distributed widely in Africa and Madagascar, with one species on the Arabian peninsula and one in India. Generally evergreen trees and shrubs, they are distinct from other African genera in the Rutaceae due to their digitately (1-)3(5-) foliate leaflets and their unarmed stems. Most species can be found in tropical lowland to submontane forest, with a few found in drier habitats. *Vepris* species are also used as indicators of healthy, relatively undisturbed forests as they are not known to be pioneers (Cheek et al., 2019).

Like other members of Rutaceae, *Vepris* species are characterized by gland dots on leaves that are filled with aromatic compounds. Many species are also known to have important secondary metabolites in root and stem tissue (Ombito, Chi & Wansi, 2021). The secondary metabolites in these tissues are utilized all over Africa in traditional medicine (Ombito, Chi & Wansi, 2021). The compounds produced are used in various forms to treat a large number of ailments, from everyday problems such as wounds and sores, to more long lasting issues such as rheumatic pains, infertility, and malaria (Ombito, Chi & Wansi, 2021). A recent review reports that 213 compounds have been isolated from various *Vepris* species, including alkaloids, quinolones, terpenoids, triterpenoids, flavonoids, and coumarins (Ombito, Chi & Wansi, 2021). Some of these compounds have been tested for bioactivity and have displayed antimicrobial, cytotoxic, anti-protozoal, and insecticidal properties (Mwangi et al., 2010; Langat, 2011; Atangana et al., 2017; Ombito, Chi & Wansi, 2021; Ojuka et al., 2023). These properties make the genus a promising one for pharmaceutical research.

The genus underwent a major taxonomic rearrangement in the 1990s. Mziray (1992) collapsed the genera *Araliopsis* Engl.*, Diphasia* Pierre*, Diphasiopsis* Mendonça, *Oricia* Pierre, *Teclea* Delile, and *Toddaliopsis* Engl. into *Vepris* based on morphological analysis. This reorganization was later confirmed with molecular work done by Morton (2017). However, in Morton’s analysis, species were not well delimited and a better supported and more complete tree would be desirable. In subsuming six genera into *Vepris,* Mziray transferred the names of 31 species, most of which were from the former genus *Teclea.* One such species was *V. amaniensis* (Engl.) Mziray; described (as *Teclea amaniensis* Engl.) in the Flora of Tropical East Africa as a glabrous shrub with unifoliolate or occasionally 2–3 foliolate leaves, elliptic leaflets with a short broad acumen, numerous gland dots on lower leaflet surfaces, terete or occasionally winged petioles, and glabrous or pubescent inflorescences (Kokwaro, 1982). However, in a taxonomic review of unifoliolate African *Vepris,* it was found that many of the specimens ascribed to *V. amaniensis* were disparate from the few that agreed with the protologue of *Teclea amaniensis* (Cheek & Luke, 2023).Very recently, the description of *V. amaniensis* has been amended to better match the protologue and so is defined as being completely glabrous, having terete to canaliculate petioles, and always being unifoliolate (Cheek & Luke, 2023). This new delimitation has been utilized here to study the c. 30 specimens, collected from Kenya and Tanzania, that were found to disagree with the *Teclea amaniensis* protologue.

This study aimed to determine how many distinct taxa reside within this group of specimens. Morphological, biochemical, and molecular methodologies were used to address this question.

## Materials and Methods

### Morphology

We studied twenty-nine herbarium specimens from the herbarium at the Royal Botanic Gardens, Kew (K) that were previously filed as *Vepris amaniensis* but were reconsidered here as possibly distinct following an inventory of the available specimens at K by Cheek. All the specimens were collected in the East Usambara, West Usambara, Nguru, and Uluguru Mountains of Tanzania, or from the south eastern coast of Kenya. Measurements of vegetative and floral traits were taken with a ruler or a Leica S6E microscope using a graticule eyepiece measuring to 0.025 mm at maximum magnification. Where appropriate fruit or floral material was available, dissections were performed after rehydration and were photographed under a Leica M165 C dissecting microscope. Measurements of floral parts were taken from these photos using ImageJ (Schneider, Rasband & Eliceiri, 2012). The specimens were sorted into groups based on two distinctive vegetative character states: the absence or presence of winged petioles, and the number of leaflets per leaf. These traits resulted in four groups; winged petiole with unifoliolate leaflets (WU), winged petioles with 1–3 foliate leaflets (WT), canaliculate petioles with unifoliolate leaflets (GU), and canaliculate petioles with 1–3 foliate leaflets (GT). The GU group was then split into two subsections, one with proportionally narrower leaflets (GU lance) and one with proportionally broader leaflets (GU broad), to account for two specimens with distinctively narrower leaflets. Specimens studied, their morphological groupings, biochemical sampling, and GenBank accessions can be found in Table 1.

**Table 1:** Herbarium samples and GenBank accessions. All herbarium specimens cited were seen and housed at K. Where samples were pooled to make one biochemical extract they are marked as combined. All specimens were sampled for DNA sequencing.

Morphological group	RBG Kew Herbarium specimen	Biochemistry sampling, mass (g)	Sample name	ITS GenBank accession	trnL-trnF GenBank accession
GT (Grooved petioles, 1–3 foliate leaflets)	R.B. Drummond and J.H. Hemsley 3456				
	Ruffo and Mmari 2354	0.751	GT(1)	OR470720	
	Luke & al 5242				OR466960
	Borhidi et al. 85240				OR466963
	Jon Lovett 263			OR470725	OR466958
	Andrew R. Marshall 1423	combined 0.8674	GT	OR470727	PP328477
	R.M. Polhill, J.M. Lovett 5007	combined 0.8674	GT		PP328476
	Ruffo and Mmari 2170				OR466965
GU lanceolate (grooved petioles, unifoliolate lanceolate leaflets)	Ruffo and Mmari 2306	0.7843	GU5	OR470718	OR466968
	Ruffo and Mmari 1785				
GU broad (Grooved petioles, unifoliolate broad leaflets)	Ruffo and Mmari 2304			OR470717	OR466966
	Ruffo and Mmari 2243	0.6327	GU4	OR470732	
	P.J. Greenway 4895				
	E.B. Wallace 939				OR466971
WT (Winged petioles, 1–3 foliolate leaflets)	Luke and Robertson 1716				
	Luke and Robertson 5848			OR470731	OR466964
	D. Napper 1380			OR470729	
	Robertson and Luke 4538			OR470730	OR466959
	R.B. Drummond and J.H. Hemsley 3802	0.6872	WT(2)		OR466955
	B.Mohoro UMBCP 329			OR470728	OR466962
	Kisena 1631	combined 1.8253	WT	OR470724	OR466957
	S.P. Kibuwa 5459	combined 1.8253	WT	OR470723	OR466956
	Ruffo and Mmari 1724	combined 1.8253	WT		OR466967
	Ruffo and Mmari 2307			OR470719	OR466969
WU (Winged petioles, unifoliolate leaflets)	S. Paulo 168				
	S.R. Semsei 1508			OR470721	OR466970
	Mgaya 157	0.3254	WU(T)		
	Drummond and Hemsley 1101			OR470722	
	Luke and Robertson 1900	0.8317	WU(K)		
	Luke WRQ 18906, unmounted specimen from Kenya	6.3125	VAK	OR470726	OR466961
Samples for DNA only					
*Vepris amaniensis*	J. Lovett, India Ellis and Alison Keeley 869			OR470733	OR466972
*Vepris sansibarensis*	T. Muller 4088			OR470736	OR466976
*Vepris sp. A* (from Cabo Delgado)	J.E. Burrows & S.M. Burrows			OR470737	OR466977
*Vepris sp. nov.* (from Mabu mt.)	F. Dowsett-Lemaire 2529			OR470735	OR466975
*Vepris trichocarpa*	Ian Derbyshire 1072			OR470738	OR466978
*Vepris fischeri (Vepris trichocarpa)*	J Timberlake 5948				OR466973
*Vepris hemp*	Hemp 7152			OR470734	OR466974

Mapping of specimens was done with coordinates directly as recorded, or with a combination of locality data and the Index of Collecting Localities for the Flora of Tropical East Africa (Polhill, 1988). Mapping was done with ArcGIS Pro (2021).

The electronic version of this article in Portable Document Format (PDF) will represent a published work according to the *International Code of Nomenclature for algae, fungi, and plants* (ICN [Shenzhen Code]; Turland et al., 2018), and hence the new names contained in the electronic version are effectively published under that Code from the electronic edition alone. In addition, new names contained in this work which have been issued with identifiers by IPNI will eventually be made available to the Global Names Index. The IPNI LSIDs can be resolved and the associated information viewed through any standard web browser by appending the LSID contained in this publication to the prefix “http://ipni.org/”. The online version of this work is archived and available from the following digital repositories: PeerJ, PubMed Central SCIE, and CLOCKSS

### Chemistry

Samples were collected from well preserved and representative herbarium specimens within the morphological groupings, as well as one dried sample sent directly from Kenya (*Luke WRQ* 18906) which was included due to the large amount of its mass that could be sacrificed for extraction. Material was ground to a powder in a spice grinder and/or a pestle and mortar. The powder was extracted in methylene chloride (CH_2_Cl_2_ abbreviated DCM) overnight, vacuum filtered and washed, and then extracted in methanol (MeOH) over the next night. Purification, analysis, and characterization of compounds were done with liquid chromatography, thin plate chromatography (TLC), nuclear magnetic resonance spectrometry (NMR), and high-resolution mass spectrometry (HR-MS).

Separation of compounds for each extraction was done using a 2 cm diameter column packed with silica (40–63 micron Davisil^®^). Solvent systems and the corresponding compounds eluted are outlined in Table 2. Extracts yielded seven compounds; compound **1** was determined to be tecleanthine (Atangana et al., 2017), compound **2** to be evoxanthine (Ombito, Chi & Wansi, 2021), **3** to be 6-methoxytecleanthine (Atangana et al., 2017), **4** to be tecleanone (Casey & Malhotra, 1975), **5** to be 1-(3,4-methylenedioxyphenyl)-1,2,3-propanetriol (Rahman & Moon, 2007), **6** to be lupeol (Ombito, Chi & Wansi, 2021), and **7** to be arborinine (Langat, Kami & Cheek, 2022).

**Table 2:** Extraction solvent systems. Solvent systems and the chemicals found at each stage. Methylene chloride is abbreviated to DCM.

	**Solvent system**	**Compound isolated in fraction**
1. DCM extracts	Hexane	
	Hexane:DCM 1:1	
	Hexane:DCM 2:80	Lupeol (**6**)
	DCM	Tecleanone (**4**)
	5% EtOAc in DCM	Arborinine (**7**), 1-(3,4-Methylenedioxyphenyl) -1,2,3-propanetriol (**5**)
	10% EtOAc in DCM	
	20% EtOAc in DCM	Tecleanthine (**1**)
	30% EtOAc in DCM	Evoxanthine (**2**), 6-methoxytecleanthine (**3**)
2. Methanol extracts	DCM	
	5% EtOAc in DCM	
	10% EtOAc in DCM	Tecleanthine (**1**), evoxanthine (**2**)
	1% MeOH in DCM	
	2% MeOH in DCM	
	3% MeOH in DCM	
	10% MeOH in DCM	
	20% MeOH in DCM	

Fractions were tested for purity using aluminum-backed TLC plates (silica gel 60 F254; Sigma-Aldrich, St. Louis, MI, USA). Visualization of the plates was done with UV radiation at 245 nm, an anisaldehyde spray reagent (1% *p*-anisaldehyde: 2% H_2_SO_4_: 97% cold MeOH), and heat. Where fractions were not sufficiently pure a second smaller separation was conducted. 1D and 2D NMR data was recorded from a Bruker 400 MHz Advance NMR instrument at room temperature in either CDCl_3_ or CD_3_OD. Chemical shifts (δ) are expressed in ppm with reference solvent peaks in H^1^ and ^13^C NMR spectra placed at δ_H_ 7.26 ppm and δ_C_ 77.23 ppm for CDCL_3_ and δ_H_ 3.31 ppm and δ_C_ 49 ppm for CD_3_OD. Where samples were small and crude extract spectra were near identical within morphological groups, samples were combined to produce a stronger signal for easier chemical characterization (Table 1). The two WU herbarium samples were not pooled despite their small mass due to their distant geographical origins.

Compounds **1, 2,** and **3,** isolated from *Luke WRQ* 18906 were dissolved in MeOH and confirmed by mass on a Thermo Scientific Orbitrap fusion mass spectrometer (Thermo Fisher Scientific, Waltham, MA, USA). Compound **5,** also isolated from *Luke WRQ* 18906, could not be confirmed with MS, and the remaining compounds could not be isolated in sufficient quantity or quality to be analyzed.

### DNA

The sampling and methods used here are informed by those used in Morton (2017). All samples studied for morphology were sampled for DNA, as well as several other *Vepris* species for comparison (Table 1). Extractions were carried out with 20–30 mg of leaf material ground in a Mixer Mill until powdered. Samples were incubated for 30 min in a 65 °C isolation buffer solution of CTAB (747 µL) and 2-mercaptoethanol (3 µL). SEVAG solution (750 µL, CHCl_3_: isoamyl alcohol = 24:1) was added and samples were shaken in an orbital shaker at 250 rev/min for 30 min and then centrifuged at 13,000 rpm for 15 min. The supernatant of each sample was transferred into new tubes with 500 µL isopropanol and stored at −20 °C for three days. The samples were then centrifuged for another 15 min, after which the aqueous phase was decanted, and the pellet washed with 70% ethanol twice, with 15 min of centrifuging between washes. The pellets were allowed to dry overnight at room temperature and then resuspended in 100 µL of water. A 1% agarose gel with ethidium bromide was then loaded for quality check.

Two markers were sequenced, the nuclear transcribed spacer (ITS) region and the plastid trnL-F intron and spacer. For ITS primers 101, 102, 2, and 3 were used. PCR Methodology followed Sun et al. (1994). PCR of the whole region (primers 101–102) was attempted, as well as from the combinations 101-2 and 3-102 to maximize likelihood of successful sequencing. The PCR solution was 25 µL consisting of 1µL sample DNA, water (5 µL), TBT (5 µL), Dream Taq (12.5 µL, Thermo Sci, 4 mM MgCl_2_), DMSO (2%, 0.5 µL) and 0.5 µL of each primer (0.2 µM). PCR conditions were an initial denaturation and activation for 2 min at 94 °C, then 28 cycles of denaturation for 1 min at 94 °C, annealing for 1 min at 52 °C, and one minute of extension at 72 °C. A final 7-minute extension at 72 °C completed the process.

For trnL-F we used the primers c and f following the method of Taberlet et al. (1991). Once again PCR of the whole region was attempted, with c-d and e-f also performed to maximize likelihood of success. The PCR solution was 25 µL, consisting of 1 µL sample DNA, water (5.5 µL), TBT (5 µL), Dream Taq (12.5 µL, 4 mM MgCl_2_; Thermo Fisher Scientific), and 0.5 µL of each primer (0.2 µM). PCR conditions were an initial denaturation and activation for 2 min at 94 °C, then 35 cycles of denaturation for 1 min at 94 °C, annealing for 1 min at 50 °C, and two minutes of extension at 72 °C. A final 4-minute extension at 72 °C finished the process.

PCR products were cleaned by using a binding buffer (125 µL, Buffer PB Qiagen, Hilden, Germany) and a PCR clean-up column (NucleoSpin), centrifuging (13,000 rpm, 1 min) the solution through and then washing the column twice with 600 µL of AW1 wash buffer (Qiagen). The columns were transferred to clean tubes and 30 µL of 65 °C EB elution buffer (Qiagen) was added. After 10 min samples were centrifuged at 13,000 rpm for 1 min to draw the DNA through the column.

Cycle sequencing was performed for each successful PCR product. The mixture for ITS primers was 1 µL sequencing buffer, 0.15 µL water, 0.1 µL (2%) DMSO, 0.25 µL BigDye Premix 3.1 (Thermo Fisher Scientific), and 0.5 µL of 1 pmol/µL of corresponding primer to make 2 µL of solution. Depending on DNA concentration, 1–3 µL of PCR product was added, with water added for a final reaction volume of 5 µL. The solution for *trnL-F* primers is the same as above, except for the exclusion of DMSO. Samples were then all subject to 26 cycles of 10 s at 96 °C, 5 s at 50 °C, and 4 min at 60 °C. Products were then cleaned with NaAC and 100% EtOH, resuspended in water and Sanger sequenced on an Applied Biosystems (Waltham, MA, USA) 3730xl DNA analyzer.

### Phylogenetic analysis

Geneious Prime (Geneious Prime, 2023. 1.2) was utilized to combine complementary strands and check base-calling, produce alignments of the obtained sequences using the MUSCLE algorithm, and concatenating the resulting alignments into one matrix. Available ITS and *trnL-F* sequences for *Vepris* in GenBank were included in the alignment (Table 3), as well as two species of *Zanthoxylum* and *Fagaropsis angolensis* (Engl.) Dale to act as outgroup taxa in subsequent analysis (Morton, 2017). Seven specimens; *Ruffo and Mmari* 1785*, Greenway* 4895*, Mgaya* 157*, Luke and Robertson* 1900*, Paulo* 168*, Luke and Robertson* 1716*, and Drummond and Hemsley* 3456 failed to be amplified at the PCR stage or produced sequences too noisy to be used and so were not included in the analysis. All other specimens had at least partial sequences of either the ITS or the trnL-F markers and were included.

**Table 3:** GenBank data used in phylogenetic analysis. Voucher numbers, references, and accession numbers of DNA data pulled from GenBank.

**Samples taken from GenBank**	**Voucher number**	**Reference**	**ITS accession number**	**trnl-trnF accession number**
*Fagaropsis angolensis*	529288 CM 529290 CM	Morton (2017) Morton (2017)	KU193664.1 KU193665.1	KU193632.1 KU193633.1
*Vepris amaniensis*	RC70 529281CM	Veldman et al. (2020) Morton (2017)	MN114145.1 KU193666.1	KU193634.1
*Vepris elliotii*	Ranaivojaone 592 (US)	Appelhans & Wen (2020)	MK882482.1	K883751.1
*Vepris eugeneiifolia*	5614653 MO	Morton (2017)	KU193684.1	KU193652.1
*Vepris glomerata*	5393948 MO	Morton (2017)	KU193686.1	KU193654.1
*Vepris grandifolia*	Boyona AF 4244019 MO	Morton (2017) Morton (2017)	KU193683.1 KU193669.1	KU193651.1 KU193637.1
*Vepris heterophylla*	5518017 MO	Morton (2017)	KU193682.1	KU19650.1
*Vepris lanceolata*	5769420 MO Kew 2343	Morton (2017) Groppo et al. (2008)	KU193685.1	KU193653.1 EU853823.1
*Vepris morogoresis*	4959977 MO	Morton (2017)	KU193663.1	KU193631.1
*Vepris nobilis*	529291 CM 4598475 MO	Morton (2017) Morton (2017)	KY508613.1 KU193670.1	KY508614.1 KU193638.1
*Vepris sansibarensis*	5750529 MO	Morton (2017)	KU193681.1	KU193649.1
*Vepris simplicifolia*	NMK:EA 13545 Chase 1764 K	Mwaura (2020) Groppo et al. (2008)	MT137500.1	EU853824.1
*Vepris sp.*	MP 668	Veldman et al. (2020)	MN257824.1	
*Vepris stolzii*	5700270 MO 5315965 MO	Morton (2017) Morton (2017)	KU193680.1 KU193676.1	KU193648.1 KU193644.1
*Zanthoxylum chevalieri*	6177214 MO	Morton (2017)	KU193687.1	KU193655.1
*Zanthoxylum deremense*	5301622 MO	Morton (2017)	KU193688.1	KU193656.1

A Bayesian inference analysis was performed with MrBayes (Huelsenbeck & Ronquist, 2001, v3.2.7). The ITS and *trnL-F* regions were treated as independent partitions in the analysis. The General Time Reversible (GTR) model with a proportion of invariable site and a gamma shape to account for rate heterogeneity among sites (GTR+I+G) was assigned to both partitions. *Zanthoxylum chevalieri* P.G. Waterman was selected as the outgroup taxon because of its availability as a sister genus with relevant data on GenBank. Posterior probability distribution was estimated using Markov chain Monte Carlo sampling (MCMC) over 7 million generations, sampled every 1000th generation and saving branch length.

Tracer (Rambaut et al., 2018, v1.7.2) was used to determine if all the parameters of the analysis reached a stationary phase. A final consensus tree was compiled in MrBayes (v3.2.7) with a burnin phase of 10% (1,000 trees) and using the option contype = allcompat. The resulting tree was visualized using FigTree v1.4.4 (Rambaut, 2010).

## Results and Discussion

### Morphology

**Figure 1:**
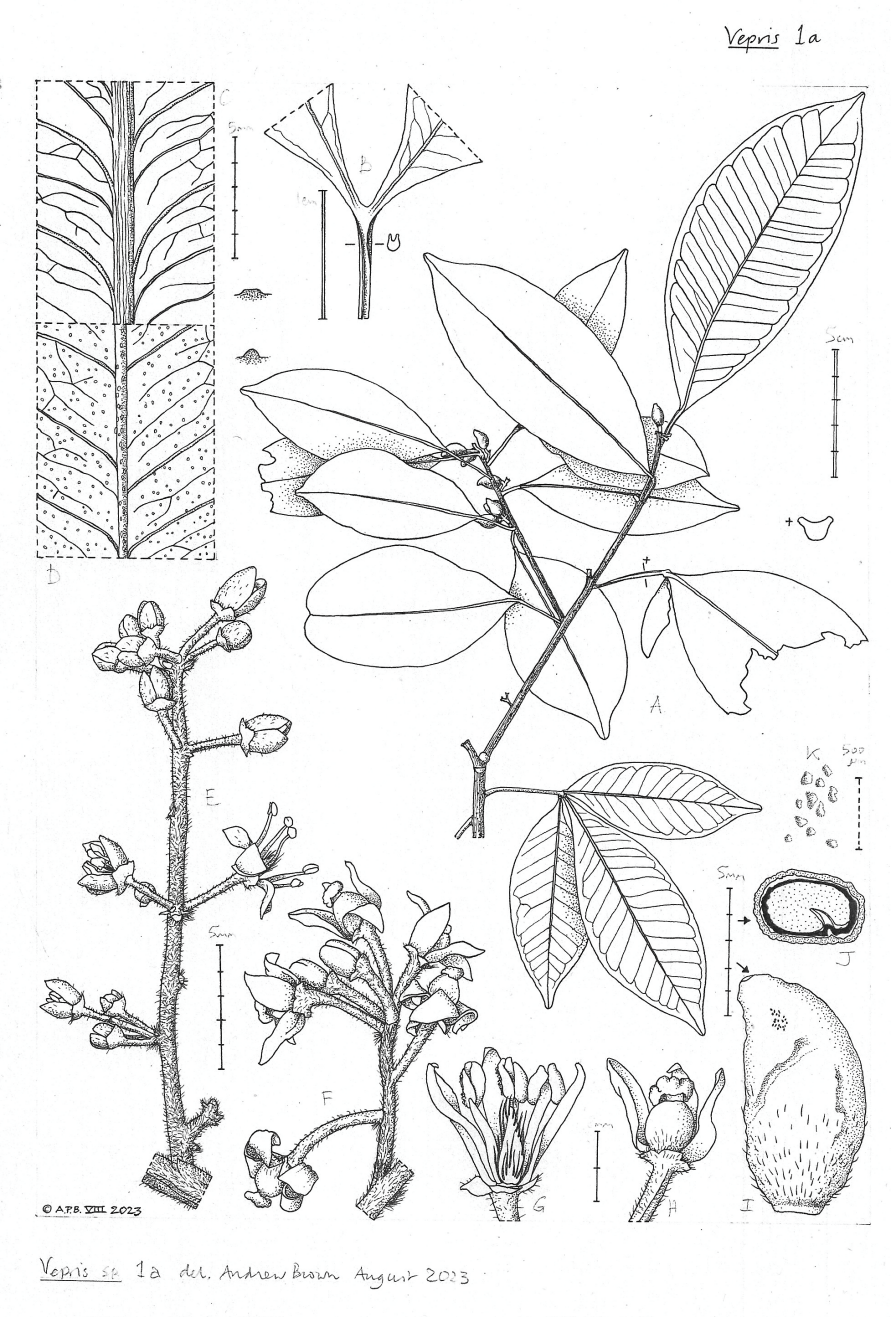
*Vepris usambarensis* plate 1. (A) Habit with top side of petiole at * (B) base of bifoliolate leaf, adaxial, with topside of petiole (C) leaf, adaxial, with profile of midrib (D) leaf, adaxial, with profile of midrib (E) male inflorescence (F) male flower after removal of one sepal and petal (G) female inflorescence (H) female flower after removal of one petal (I) mature fruit, side view (J) mature fruit top side (K) detail of pitting on fruit surface. Material used: A–D, I–K Napper 1380 (K003470010), E, F Marshall et al. 1423 (K003470011), G, H, Lovett 263 (K003470013). Image by Andrew Brown.

**Figure 2:**
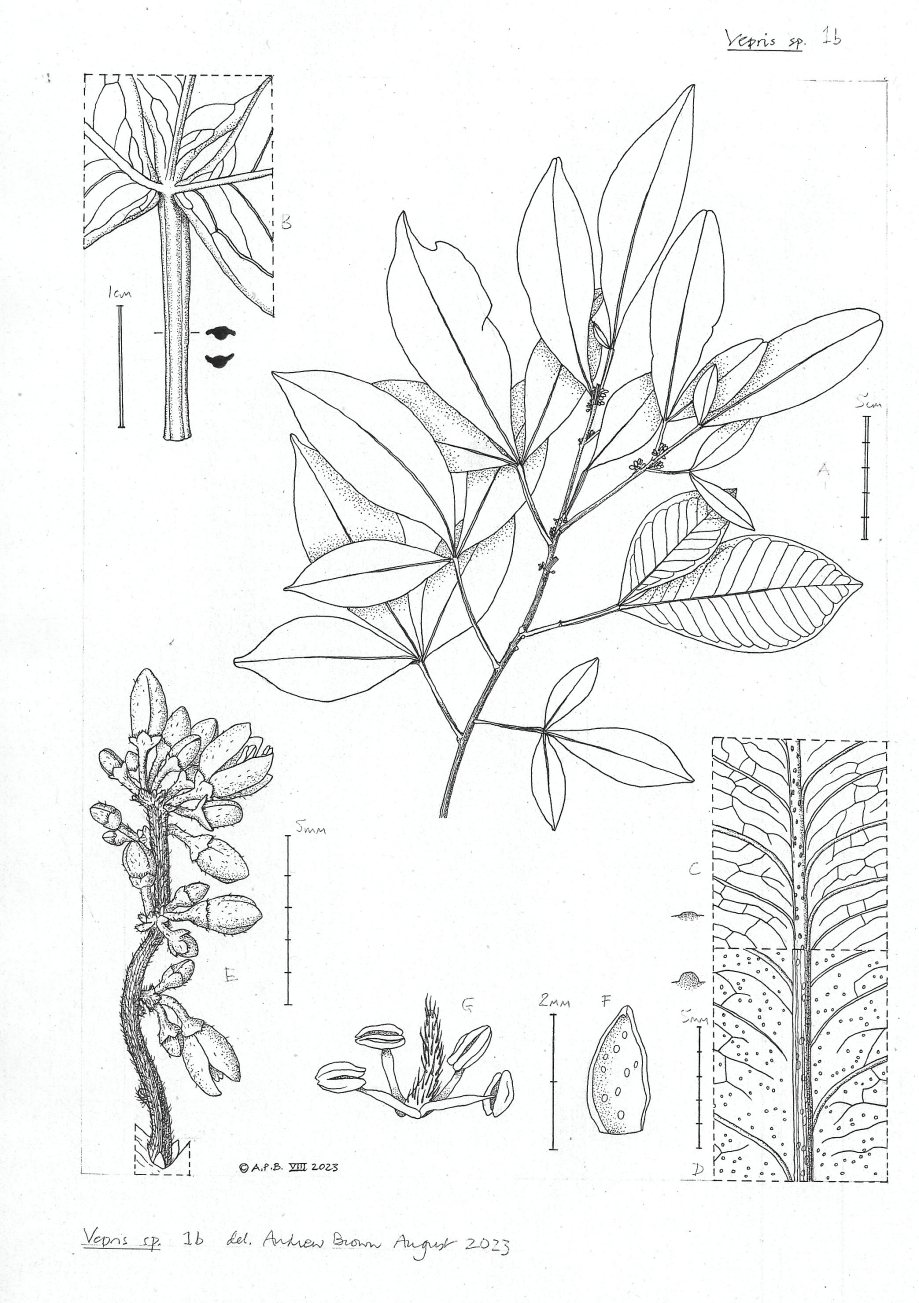
*Vepris usambarensis* plate 2. (A) Habit (B) adaxial view of trifoliolate leaf and petiole with two variants of petiole top side (C) leaf adaxial with profile of midrib (D) leaf abaxial with profile of midrib (E) male inflorescence (F) inner face of petal showing oil glands (G) stamens and vestigial ovary. Material used: A, Drummond et al. 3802 sheet 3 (K003470007), B–G, Drummond et al. 3802 sheet 2 (K003470008). Image by Andrew Brown.

*Vepris amaniensis* is delimited as being glabrous, unifoliolate, having terete petioles, and inflorescences 0.9–4(-5)cm long (Cheek & Luke, 2023). The samples measured for this study are morphologically distinct, and are separated from *V. amaniensis* by their canaliculate to winged petioles, 0.5–2.3 cm long inflorescences, 1–3 foliolate leaflets, and hairs on both inflorescences and stem apices. As such, they are here described as the new species *Vepris usambarensis* (Figs. 1 and 2).

It was originally hypothesized that there might be more than one new taxon within the specimens studied. However, this study could not generate enough support to clearly separate additional taxa other than the proposed new species *V. usambarensis.* Samples were originally separated into four groups; those with winged petioles and unifoliolate leaflets (WU), winged and 1–3 foliolate leaflets (WT), canaliculate petioles and unifoliolate leaflets (GU), and canaliculate and 1–3 foliolate leaflets (GT). These two characters, petiole morphology and leaflet number, were the only distinctive characters by which the samples could be divided. All other characters, both vegetative and reproductive, appeared to have too little variation across specimens to be credibly utilized in differentiating groups. The availability of mature flowering structures in this study group was low and so with more complete resources, distinct reproductive characters may become apparent.

The use of leaflet number as a delimiting character proved fairly weak across specimens; many nearly exclusively expressing unifoliolate leaflets only to have one or two multi-foliolate leaflets, or vice versa. Others had a relatively even mix of one, two, and three foliolate leaflets. The plasticity in leaflet number displayed on a single specimen suggests that even on specimens that only included unifoliolate material, it was not possible to rule out a higher number of leaflets elsewhere on the plant from which these specimens were taken. In *Vepris*, petiole morphology and leaflet number are generally important characters used in delimiting species (Cheek & Luke, 2023), and species are generally able to be defined by a single leaflet and petiole state. That this species has so much variation makes it an interesting one for further taxonomic and phylogenetic study. More reproductive specimens and further DNA sequencing may also prove the presence of more taxa.

This variability of leaflet number poses a question concerning the evolution and ecological relevance of having a single leaflet *versus* multiple leaflets. That these plants have and express the genes for both could suggest a maximizing of efficiency by using two states with differing benefits, or it could simply be an evolutionary artifact going from one state to another.

With support for leaflet number as a taxon identifier low in this case, demarcating additional taxa solely on petiole morphology could not be justified, and so here the study specimens are all classified together as a new, unusually morphologically variable species separate from *V. amaniensis*.

**Vepris usambarensis** *Ciam. & Cheek,* sp. nov. Holotype: Tanzania, Lushoto district, Mazumbai, West Usambaras, University Forest reserve, 1,480 m, fr, fl, March 1984, *Jon Lovett* 263 (estimated 4.8°S, 38.5°E, herbarium specimen, K003470013).

*Dioecious evergreen shrub* 0.5–2.5(-5)m tall, alternate branching, grey bark, internodes 0.45–5.3 cm long, stem diameter at lowest leafy node (1-)1.5–5 mm, puberulent at stem apex, rapidly becoming glabrous within 4 nodes of the stem apex, lenticels sub elliptic, raised, c. 0.3–0.5(−0.8) by 0.2–0.3 mm, coverage up to 40% of surface area after 7 nodes from stem apex, leaflets 1–3 foliolate (where a plant is predominantly 1 or 3 foliolate there will generally be at least one leaflet displaying a higher or lower leaflet number).

*Leaflets* elliptic, 5.8–17.7(-22.7) cm long, c. (2.2-)2.8–5(-7) cm wide, margin simple, entire, where 2–3 foliolate, lateral leaflets are overall 40–60% more reduced than the median; leaflets acuminate, acumen 0.3–1.9 cm long, 2.5–7 mm wide; secondary veins 13–22 on each side of midrib, brochidodromous, gland dots conspicuous on abaxial side, clear in transmitted light, pale green in reflected light, c. 0.1 mm diameter, density 5–7(-11) dots/mm^2^.

*Petiole* (0.4-)0.9–3.6(−5.1) cm long, articulated at top and bottom, base puberulent when it falls within 4 nodes of stem apex, apex and abaxial surface with occasional hairs, hairs simple, thickened pulvinus at apical articulation sometimes present, canaliculate to winged, wings up to c. 2.4(-4) mm wide at apex.

*Petiolule* 0.1–0.3(−0.5) cm long, inconspicuous, terete, glabrous.

*Inflorescences* axillary paniculate, 0.5–2.3(−3.8) cm long, all parts densely hairy when young, becoming less dense with age, hairs simple, patent, 0.02–0.07 mm long, yellow upon drying.

*Peduncle* 0.1–0.3(−0.7) cm long, sparsely hairy to puberulent, hairs simple.

*Rachis* 0.5–1.1(−1.5) cm long, sparsely hairy to puberulent, hairs simple, internodes 0.1–0.5 cm long, alternate, node bearing 1–3 flowers.

*Bracts* four lobed, lobes triangular, c. 0.25 x 0.5 mm, cupuliform, resembling sepals, surrounding peduncle, puberulent when young, becoming sparsely hairy with age, hairs simple, not seen intact on mature flowers.

*Bracteoles* subtending pedicels, as for bracts.

*Pedicel* 1.3–1.8 mm long, sparsely puberulent, hairs simple, yellow.

*Flowers* dioecious, male and female both 2.3­-3.3 mm long.

*Sepals* four, triangular, c. 0.6 mm long x 0.8 mm wide, united from base to c. one third of length, ciliate, occasional gland dots.

*Petals* four, elliptic, c. 2–2.4 mm long, 0.9–1.39 mm wide, drying golden yellow, thickened tip, petal becomes fully reflexed with age, occasional persistent hairs on outer surface, gland dots present on bud, clustered near apex, drying yellow.

*Stamens* in male flowers 4(-5), filaments 2–2.7 mm long, dorsiventrally flattened, tapering at top, anthers ovoid to discoid, diameter 0.46–0.59 mm, medifixed. Staminode remnants observed in available female flowers.

*Ovary* in male flowers vestigial, cone shaped, 0.83–1 mm long, c. 0.34–0.38 mm wide at base, densely covered in semi-appressed simple yellow hairs c. 0.5–0.6 mm long, unilocular, yellow-orange, slightly lobed at bottom around stamens (suspected vestigial disk). In mature female flowers ovary is sub-ovoid, c. two mm long and 1.4 mm wide, unilocular, vesicular, scabrid near base, drying brown, 4–5 orange lobes near base around where staminodes emerge, stigma discoid, c. 1.4 mm diameter, convex, style minute, c. 0.1 mm long.

*Fruit* a single-seeded berry, ellipsoid to apex slightly beaked, 9–13 mm long x 3–8 mm wide, thinly fleshy, exocarp c. 0.4 mm thick, ripening green, brown purple on drying, 4–5 orange lobes generally persistent on bottom, conspicuous gland dots, yellow to brown, slightly raised, 0.1–0.26 mm diameter, occasional persistent hairs, pedicel accrescent, 2–6 mm long.

*Seed* tan, ellipsoid, dimensions slightly smaller than in fruit, single longitudinal groove.

**Representative specimens examined:** (All specimens were seen and housed at K)

Tanzania, Morogoro Rural dist, Mkungwe forest reserve, fr, fl, Aug 13, 2000, *B. Mhoro UMBCP* 329 (est 6°53′S 37°55′E, K003470017),

Tanzania, Muheza dist, Amani-Kwamkoro road 2miles SE of Amani, fr, July 15, 1953, *R.B. Drummond and J.H. Hemsley* 3456 (estimated 5°7′S 38°37′E, K003470026),

Tanzania, Muheza dist, Kwamkoro forest reserve, Monga, fr, July 18, 1986, *Ruffo and Mmari* 2354 (est 5°06′S 38°37′E, K003470022),

Tanzania, Muheza dist, east Usambara mt, Kwamkoro forest trail 3, fl, March 1998, *Luke & al.* 5242 (est 5°10′S 38°36′E, K003470027),

Tanzania, Muheza dist, fl, fr, June 28, 1987, *Ruffo and Mmari* 2170 (est 5 °10′S 38°48′E, K003470023),

Tanzania, Muheza dist, Kilanga forest reserve, fr, Aug 24, 1986, *Ruffo and Mmari* 1785 (est 5°18.5′S 38°38′E, K003470020),

Tanzania, Muheza dist, Kwamkoro forest reserve, fr, July 1, 1985, *Ruffo and Mmari* 2243 (est 5°10′S 38°36′E, K003470030),

Tanzania, Muheza dist, Mgue Sangerawe, fl, Oct 2, 1937, *P.J. Greenway* 4895 (est 5°8′S 38°37′E, K003470029),

Tanzania, Muheza dist, Mtai forest reserve, fl, Sep 13, 1996, *Kisena* 1631 (est c.4°50′S 38°46′E, K003470035),

Tanzania, Muheza dist, Kibwanda to Bulwa foot path, st (leaves), Nov 10, 1981, *S.P. Kibuwa* 5459 (est 5°3″S 38°41″E, K003470015),

Tanzania, Mvomero dist, Turiani, fr, Nov 1953, *S. Paulo* 168 (est 6°9′S 37°35′E, K003470038),

Tanzania, Mvomero dist, Mtibwa forest reserve, fr, Nov 1953, *S.R. Semsei* 1508 (est c. 6°7′S 37°39′E, K003470037),

Tanzania, Mvomero dist, Manyangu forest reserve, fl, July 1957, *Mgaya* 157 (est c. 6°07′S 37°34′E, K003470033),

Tanzania, Lushoto district, Usambara mts, Mahezangulu forest reserve, fl , Jan 24, 1985, *Borhidi et al.* 85240 (est 4°56′S 38°31″E, K003470028),

Tanzania, Lushoto dist, Mazumbai, west Usambara, university forest reserve, 4°48′S 38°30′E, fr, fl, Mar 1984, *Jon Lovett* 263 (K003470013),

Tanzania, Lushoto dist, near Mazumbai HQ, Mazumbai forest reserve, west Usambara Mts, 4°48′S 38°30′E, fl, July 3, 2008, *Andrew R. Marshall* 1423 (K003470011),

Tanzania, Korogwe dist, Ambangulu tea estate, fr, July 17, 1983, *R.M. Polhill, J.C. & J.M. Lovett* 5007 (est 5°2′S 38°23′E, K003470024),

Tanzania, Korogwe dist, West Usambaras, Ambangulu estate, st (leaves), Oct 1940, *E.B. Wallace* 939 (est 5°5′S 38°26′E, K003470031),

Tanzania, Korogwe dist, Lutindi forest reserve, fr, Aug 1989, *Ruffo and Mmari* 2306 (est c.4°53′S 38°38′E, K003470021),

Tanzania, Korogwe dist, Lutindi forest reserve, fr, Aug 1986*, Ruffo and Mmari* 2304 (est c.4°53′S 38°38′E, K003470032),

Tanzania, Korogwe dist, Lutindi forest reserve, fl , Aug 24, 1986, *Ruffo and Mmari* 1724 (est c. 4°53′S 38°38′E, K003470018),

Tanzania, Korogwe dist, fr, Jan 22, 1987, *Ruffo and Mmari* 2307 (est 5°9′S 38°28′E, K003470016),

Kenya, Kilifi dist, Kombeni reserve valley edge of Kaya Fimboni, fl, Aug 21, 1989, *Luke and Robertson* 5848 (est 3°54′S 39°36′E, K003470012),

Kenya, Kilifi dist, Pangani Rocks, fl, Aug 16, 1989, *Luke and Robertson* 1900 (est 3°51′S 39°40′E, K003470036),

Kenya, Kwale dist, Buda Mafisini forest reserve, fr, Feb 24, 1989, *Luke and Robertson* 1716 (est 4°27′S 39°24′E, K003470014),

Kenya, Kwale dist, Buda Mafisini forest reserve, fr, Nov 3, 1959, *D. Napper* 1380 (est c.4°27′S 39°24′E, K003470010),

Kenya, Kwale dist, Muhka forest, fr, Feb 19, 1987, *Robertson and Luke* 4538 (est 4°20′S 39°31′E, K003470019),

Kenya, Kwale dist, Buda Mafisini forest reserve, fl, Aug 16, 1953, *R.B. Drummond and J.H. Hemsley* 3802 (est 4°27′S 39°24′E, K003470009),

Kenya, Mwele Mdogo forest, Shimba Hills 12 miles SW of Kwale, fr, Feb 4, 1953, *Drummond and Hemsley* 1101 (est 4°18′S 39°21′E, K003470034).

**Figure 3:**
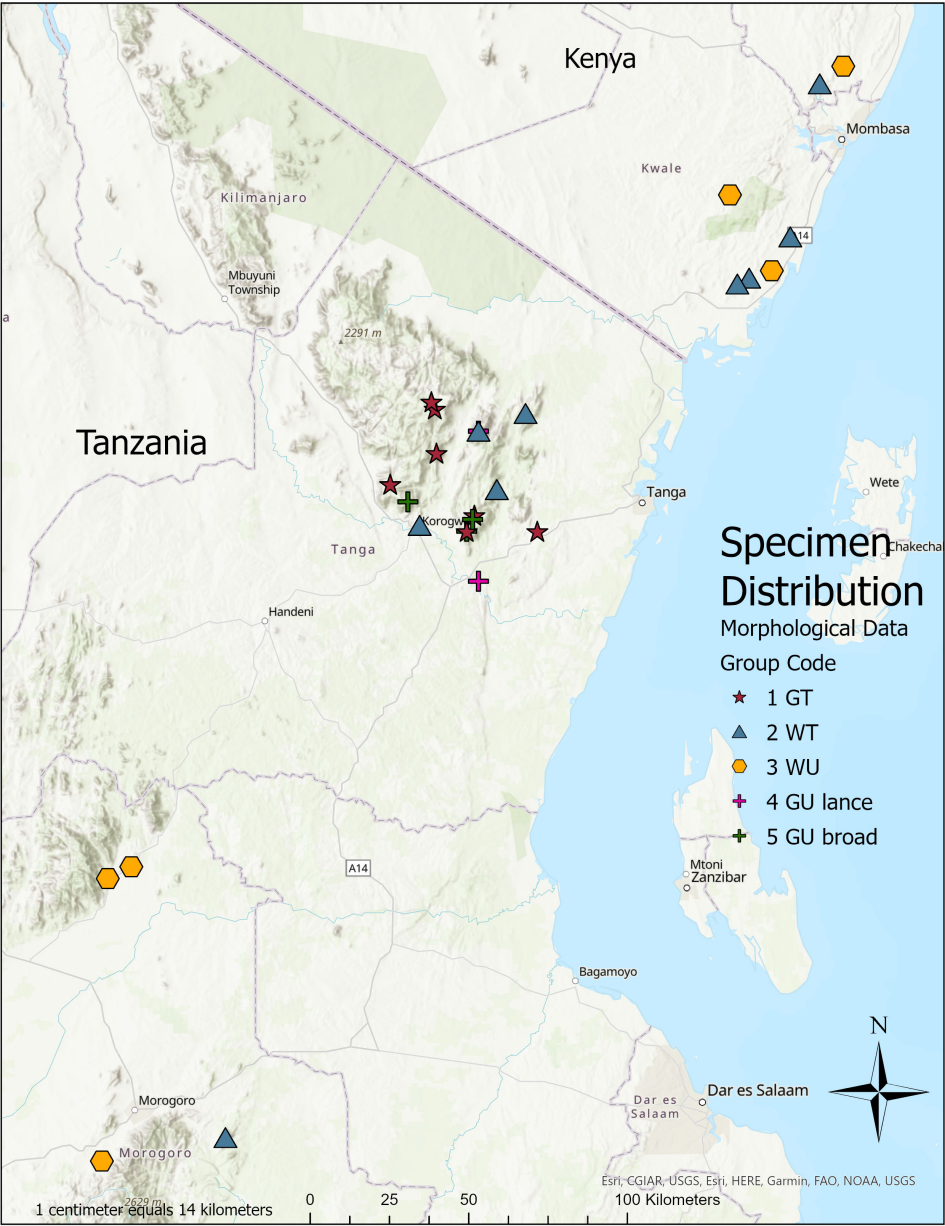
Collection sites of samples. Specimens were mapped using locality data as recorded and the Index of Collecting Localities by Polhill (1988). Produced with ArcPro. Map data: Ersi, CGIAR, USGS, HERE, Garmin, Fao, NOAA.

**Distribution:** Coastal south-eastern Kenya, East and West Usambara mountains, Nguru mountains, and the Uluguru mountains of Tanzania. (Fig. 3.)

**Habitat:** Restricted to relatively undisturbed habitat, in Tanzania relegating it to submontane to montane evergreen tropical forest. Kenyan specimens are also found in protected areas, though at much lower elevations.

**Etymology:** Named after the Usambara mountains, where the majority of the specimens were collected.

**Phenology:** Flowers March to September, and fruits August to February.

**Recognition:**

*Vepris usambarensis* can be distinguished from *Vepris amaniensis* Engl. by its canaliculate to winged petioles, the presence of an indumentum at stem apices and on inflorescences, generally shorter panicles (0.5–2.3(-3.8) cm long), and 1–3 foliolate leaflets. *Vepris amaniensis* Engl. has terete to canaliculate petioles, glabrous stems and inflorescences, 0.9–4(-5) cm long panicles, and unifoliolate leaflets (Table 4). Representative *V. amaniensis* specimens, including the neotype: Tanzania, Muheza dist, Amani, fl., May 4, 1922, *Salmon G 6171* (K000593352!; isoneotype EA) (Cheek & Luke, 2023) were consulted. Specimen information can be found in the supplemental files. Illustrations of representative *V. usambarensis* specimens are given in Figs. 1 and 2.

**Table 4:** Distinguishing characters for *V. amaniensis* and *V. usambarensis*.

Character	*V. usambarensis*	*V. amaniensis*
Petiole	Canaliculate to winged	Terete at base to canaliculate at apex
Indumentum	Puberulent at stem apices and on bracts and sepals	Glabrous
Inflorescence	0.5–2.3(−3.8) cm long	0.9–4(-5) cm long
Leaflet number	1–3 foliolate	Always unifoliolate

Note: Although the neotype of Vepris amaniensis (Engl.) Mziray is stated in Cheek & Luke (2023) to be “Salmon 171”, inspection of the annotated neotype sheet at K (see photo in Supplemental Information) shows that a digit is missing, the correct number is in fact 6171. There is no confusion of the specimen since it is annotated as the neotype by the first author of Cheek & Luke (2023) who is also the 3rd author of the current paper, and other details are the same. In addition, the specimen is (correctly) referred to in the Notes of Vepris amaniensis as “Salmon G 6171”.

### Chemistry

**Figure 4:**
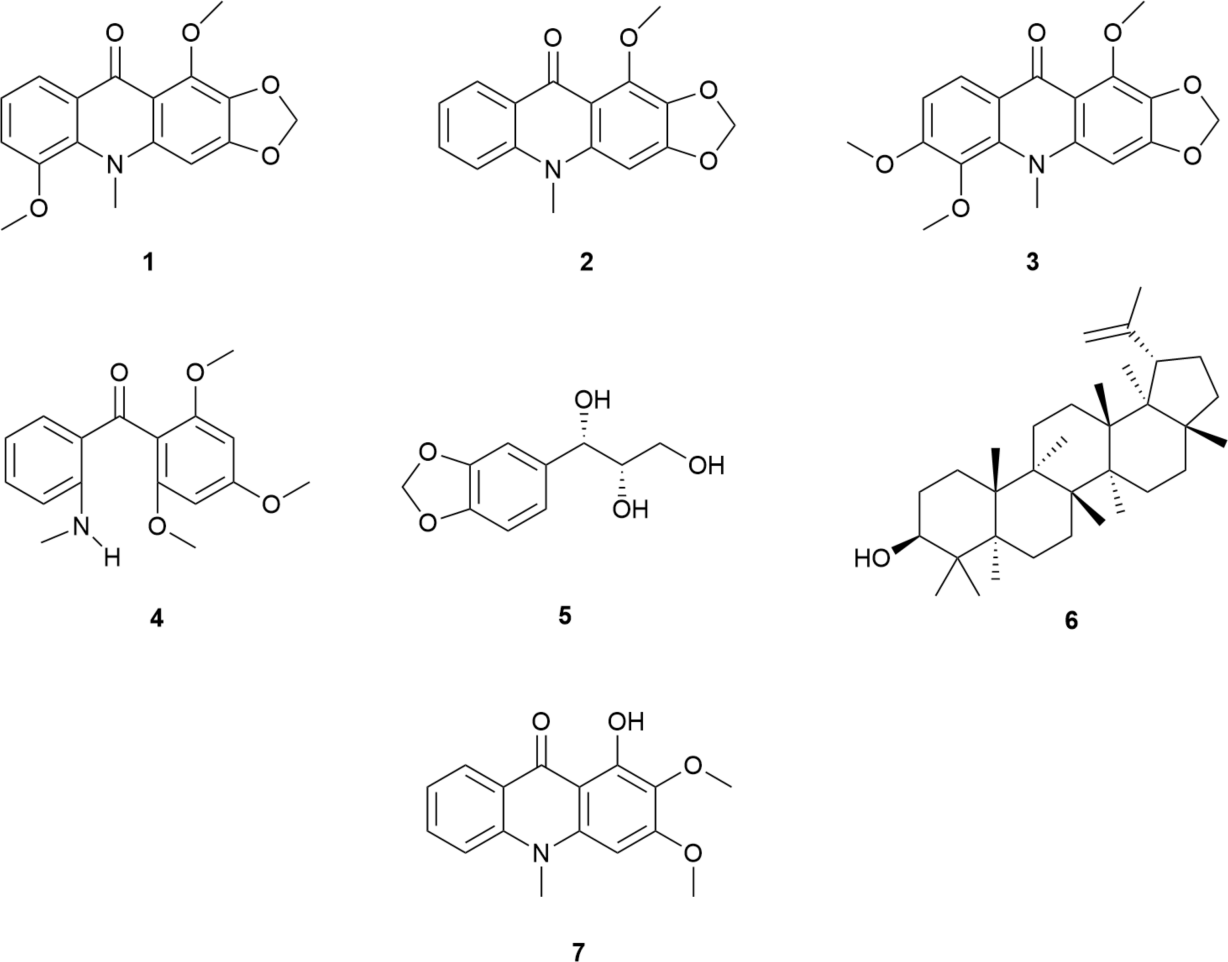
Extracted and characterized compounds. Compounds found in DCM extracts. **1.** Tecleanthine **2.** Evoxanthine **3.** 6-methoxytecleanthine **4.** Tecleanone **5.** 1-(3,4-Methylenedioxyphenyl)-1,2,3-propanetriol **6.** Lupeol **7.** Arborinine.

Of the specimens that were sampled, seven compounds were found in high enough concentration to be described; tecleanthine (**1**), evoxanthine (**2**), 6-methoxytecleanthine (**3**), tecleanone (**4**), 1-(3,4-methylenedioxyphenyl)-1,2,3-propanetriol (synonym 3′,4′-methylene ether) (**5**), lupeol (**6**), and arborinine (**7**) (Fig. 4). All compounds described were found in CH_2_Cl_2_ extracts, methanol extracts yielded tecleanthine (**1**) and evoxanthine (**2**) as well, but any other compounds were too dilute to be discerned.

**Figure 5:**
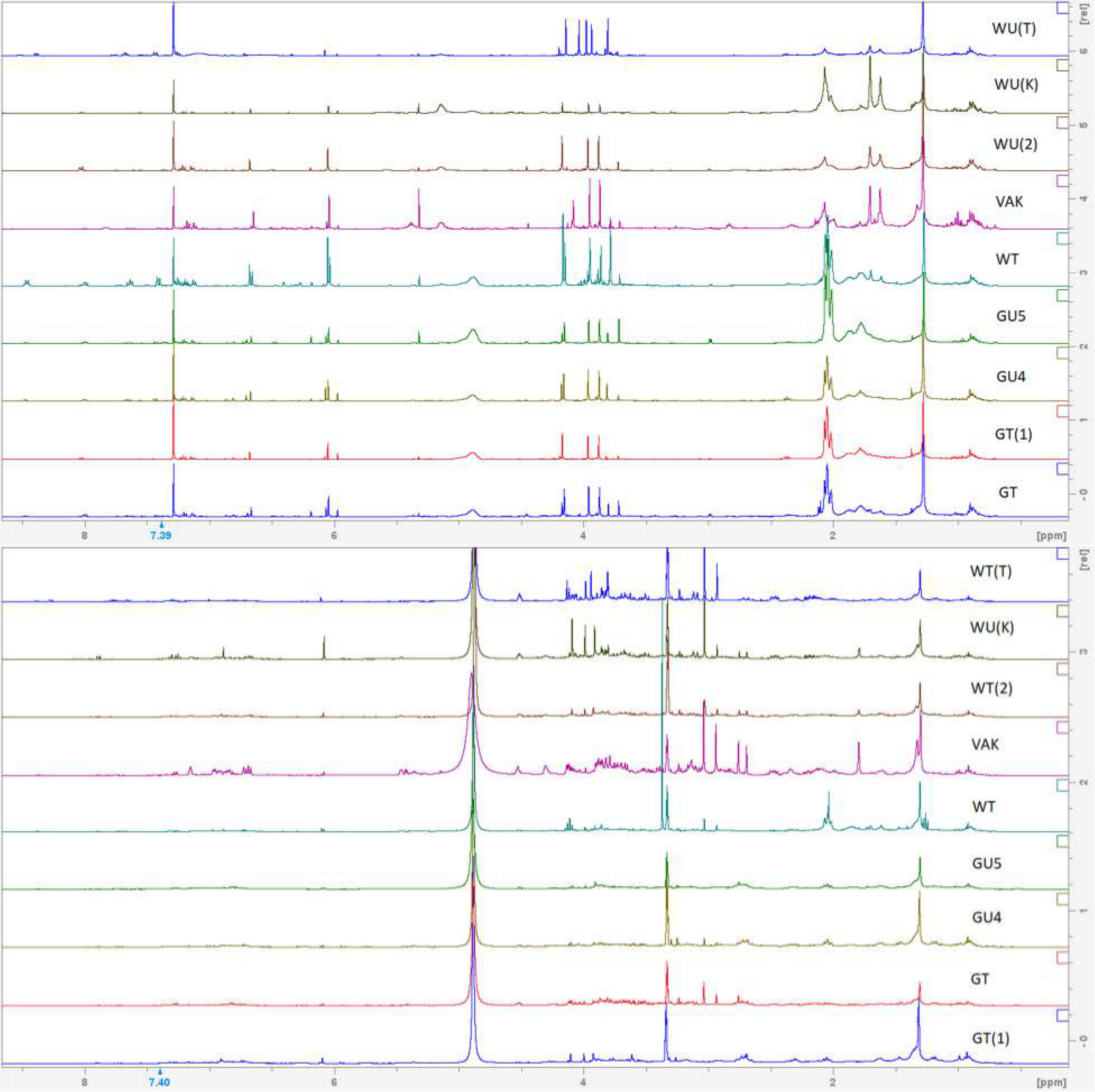
NMR spectra of DCM extracts. H^1^ NMR spectra of crude CH_2_Cl_2_ extracts.

**Figure 6:**
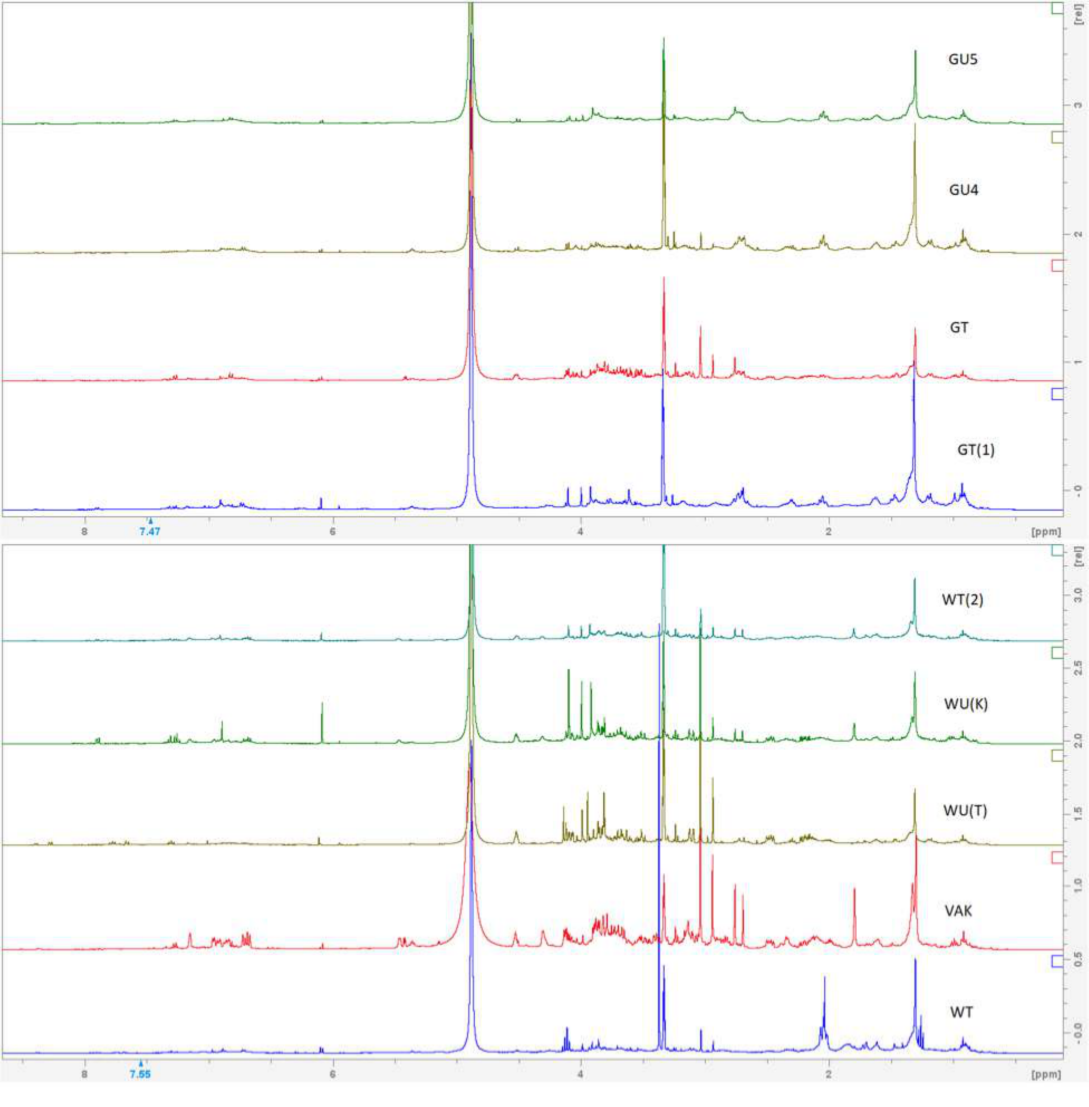
NMR spectra of methanol extracts. H^1^ NMR spectra of crude MeOH extracts.

As with the attempt to create morphological groupings within the studied specimens, the NMR profiles of the samples were too similar to each other to lend credible support to more than one taxon being present (Figs. 5–6). All samples had tecleanthine (**1**) as the dominant compound, in some samples such as GT(1) and WU(K) it was nearly the only one present. Evoxanthine (**2**) was the second most dominant compound, and the rest were notably less dominant (Table 5). The only differentiation between samples were the varying concentrations of compounds, which can be attributed to life stage, time of collection, and local climate, or the absence of very minor ones. Absences could be due to the small sizes of some samples. Minor compounds like lupeol (**6**) and arborinine (**7**) were able to be isolated from *Luke WRQ* 18906 (sample name VAK) and not others likely due to it being over six times the mass of many of the other samples.

The exact combination of chemicals found in this study are not reported in any other *Vepris* species studied for biochemistry, though all but one of the compounds identified is known to occur in the genus. *Vepris grandifolia* (Engl.) Mziray and *Vepris trichocarpa* (Engl.) Mziray, both species that occur in tropical east Africa, are reported with similar mixtures including tecleanthine, tecleanone, evoxanthine, lupeol, arborinine, and 6-methoxytecleanthine (Ombito, Chi & Wansi, 2021). The only compound not described before from a *Vepris* species is 1-(3,4-methylenedioxyphenyl)-1,2,3-propanetriol (**5**), classed as a lignan. It was first described from extracts taken from the roots of *Dendranthema zawadskii* var. *latilobum* (Maxim) Kitam. (synonymized with *Chrysanthemum naktongense* Nakai), a temperate member of the Asteraceae native to north east Asia and used medicinally in Korea (Rahman & Moon, 2007). It is not known from any other species. That these two species from disparate clades, continents, and climates produce the same chemical seems unlikely, and while its presence was confirmed with NMR analysis, it could not be confirmed with mass spectrometry analysis. Further investigation of the presence of this lignan in this species is recommended.

Lignans and alkaloids have been recognized for their potent pharmacological potential, and are known to contain compounds important to medicine (Saleem et al., 2005; Barker, 2019; Cui et al., 2020). Tecleanthine (**1**), evoxanthine (**2**), 6-methoxytecleanthine (**3**), lupeol (**6**), arborinine (**7**), and 1-(3,4-methylenedioxyphenyl)-1,2,3-propanetriol (**5**) are known to have bioactive properties including antioxidant, antiprotozoal, cytotoxic, antimicrobial, antiplasmodial, and antifeedant properties (Popp & Chakraborty, 1964; Lwande et al., 1983; Muriithi et al., 2002; Rahman & Moon, 2007; Mwangi et al., 2010; Dongfack et al., 2012; Nouga et al., 2016; Atangana et al., 2017). Tecleanone (**4**) has not been studied for any bioactivity, but it is considered an intermediary in the chemical pathway to make both tecleanthine (**1**) and evoxanthine (**2**) (Dagne et al., 1988; Singh & Bharate, 2006).

**Table 5:** Chemical components of extracted samples. Chemical compounds found in each sample. Compounds are considered major when they are readily apparent on the NMR spectrogram and are comparable in height to the reference peak. Compounds that do not meet this criteria are considered minor.

Sample	Tecleanthine	Evoxanthine	6-methoxy tecleanthine	Tecleanone	1-(3,4- Methylenedioxyphenyl) -1,2,3-propanetriol	Lupeol	Arborinine
GT	Major	Major		Minor	Minor		Minor
GU4	Major	Major		Minor	Minor		
GU5	Major	Major	Minor	Minor	Minor		
GT(1)	Major	Minor		Minor	Minor		
VAK	Major	Major	Minor	Traces	Minor	Minor	Minor
WT	Major	Major	Minor	Minor	Traces		Traces
WT(2)	Major	Major	Minor	Minor			
WU(T)	Major	Major	Minor		Minor		Minor
WU(K)	Major	Traces		Traces	Minor	Minor	Traces


### Phylogeny

**Figure 7:**
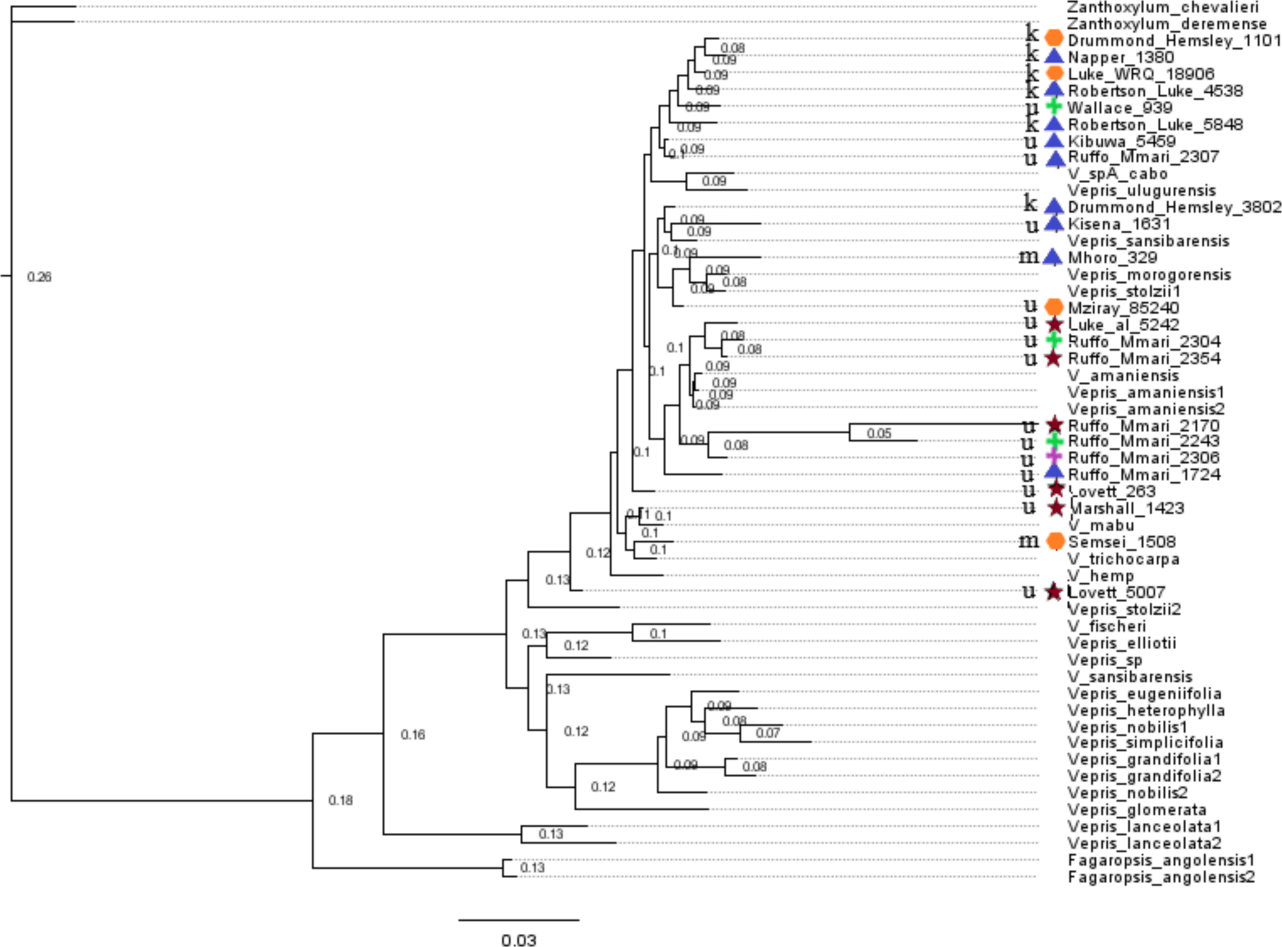
Phylogeny of both samples and GenBank data. Phylogeny produced with MrBayes. The symbols indicate the sample’s morphological group; red stars indicate GT, blue triangles indicate WT, orange hexagons indicate WU, pink crosses indicate GU4, and green crosses indicate GU5. The letter preceding the symbols indicate as follows: k for Kenya, u for the Usambara Mts, and m for the Morogoro region of Tanzania.

The use of the ITS and *trnL-F* regions here was modeled on the approach of Morton (2017), as this study is the most complete molecular work done at species level on *Vepris* to date. In Morton’s study, it is stated that discerning species with these regions is difficult, a conclusion supported here. Utilizing these two regions, isolated from each specimen studied, as well as those included from other *Vepris* species on GenBank, our consensus tree did not have any node with posterior probability over 0.26 (Fig. 7). There is a loose grouping of the specimens that were collected from Kenya, as well as a loose separation of the winged specimens from the canaliculate specimens. However, with the very small support for these relationships, these conclusions cannot be supported.

It appears that these two regions are sufficiently invariable across *Vepris* and so should not be relied upon alone in future phylogenetic analyses. Future studies in this group would require approaches that provide a greater amount of information, such as RADseq and targeted enrichment (*e.g.*, Angiosperm353; Johnson et al., 2019) to produce a better supported phylogeny and reveal any cryptic species that *Vepris* may contain.

## Conclusion

The new species *Vepris usambarensis* is here described as being distinct from *V. amaniensis*, the name under which its specimens had previously been filed. This species can be confirmed to produce tecleanthine (**1**), evoxanthine (**2**), 6-methoxytecleanthine (**3**), tecleanone (**4**), 1-(3,4-methylenedioxyphenyl)-1,2,3-propanetriol (**5**), lupeol (**6**), and arborinine (**7**). These are all chemicals known to be found in *Vepris*, save for the lignan which is known from a *Chrysanthemum* native to east Asia. The known bioactivity of both the lignan and the alkaloids present, like in many *Vepris*, give it pharmacological potential.

It was originally hypothesized that there would be more than one taxon within the study group due to the high levels of morphological variation observed in characters usually of value in differentiating species in *Vepris*. However, morphologically and biochemically any further delineations could not be supported. Molecular work was undertaken to try to add additional support; however, the chosen regions, ITS and *trnL-F*, failed to produce a tree at the species level with enough support. These regions were chosen following Morton (2017), and we conclude that in *Vepris*, they have little to no value for differentiating species.

Given the variation of morphology observed, it is recommended that further phylogenetic research be conducted with other sequencing approaches. A more complete tree of *Vepris* than the one produced by Morton (2017) with more support is desired and further investigation of the phylogenetic diversification of species *versus* morphology in the genus would shine light on the evolution and ecology of the genus as a whole.

## Supplemental Information

10.7717/peerj.17881/supp-1 Supplemental Information 1 Supplimentary table and NMR spectra Additional table for compounds detailing in which fraction they were found. Additional H1 NMR spectra of isolated compounds.

10.7717/peerj.17881/supp-2 Supplemental Information 2 DNA data for the trnL-F region of the samples studied Aligned sequences, formatted as for Genbank.

10.7717/peerj.17881/supp-3 Supplemental Information 3 DNA data for unaligned trnL-F region of two samples Unaligned samples. Formatted as for Genbank.

10.7717/peerj.17881/supp-4 Supplemental Information 4 ITS genbank accession OR470717-21

10.7717/peerj.17881/supp-5 Supplemental Information 5 ITS genbank submission OR470722-38

10.7717/peerj.17881/supp-6 Supplemental Information 6 Sample morphological data

10.7717/peerj.17881/supp-7 Supplemental Information 7 V. amaniensis neotype Image of neotype published by Cheek 2023 for Vepris amaniensis, Borhidi et al. 85340.

## Additional Information and Declarations

### Data Availability

The following information was supplied regarding data availability: The DNA sequences are available at GenBank: OR466955–OR466978, OR470717–OR470738, and PP328476–PP328477. Measurements of the herbarium specimens that are included in the article.

### New Species Registration

The following information was supplied regarding the registration of a newly described species: *Vepris usambarensis* urn:lsid:ipni.org:names:77345528-1
