# Supplementary material for: Vepris amaniensis: a morphological, biochemical, and molecular investigation of a species complex
Source: PeerJ. 2024 Sep 25;12:e17881. doi: 10.7717/peerj.17881 (PMC11438429; doi:10.7717/peerj.17881)
Supplement: Supplemental Information 1 — Additional table for compounds detailing in which fraction they were found. Additional H1 NMR spectra of isolated compounds. [file peerj-12-17881-s001.pdf]

Supplemental information

List of compounds identified and their corresponding samples/fractions. Crude signifies that the compound could be identified in the original extract without separation.

DCM extracts		
**Compound**	**Reference**	**Sample and fraction**
Tecleanthine (1)	(Atangana et al., 2017)	GT crude<br>GU5 97<br>GU4 crude<br>GT(1) crude<br>VAK 102<br>WT 33<br>WT(2) crude<br>WU(T) crude<br>WU(K) crude
Evoxanthine (2)	(Ombito, Chi & Wansi, 2021)	GT crude<br>GU5 112<br>GU4 crude<br>GT(1) crude<br>VAK 111<br>WT 33<br>WT(2) crude<br>WU(T) crude<br>WU(K) crude
6-methoxytecleanthine (3)	(Atangana et al., 2017)	GU5 136<br>VAK 118<br>WT crude<br>WT(2) crude<br>WU(T) crude<br>WU(K) crude
Tecleanone (4)	(Casey & Malhotra, 1975)	GT crude<br>GU5 32-34<br>GU4 crude<br>GT(1) crude<br>VAK crude<br>WT 28-29<br>WT(2) crude
1-(3,4-Methylenedioxyphenyl)-1,2,3-propanetriol (5)	(Rahman & Moon, 2007)	GT crude<br>GT(1) crude<br>GU5 51-55<br>GU4 crude<br>VAK 65<br>WT crude<br>WU(T) crude<br>WU(K) crude
Lupeol (6)	(Ombito, Chi & Wansi, 2021)	VAK 38<br>WU(K) crude
Arborinine (7)	(Langat, Kami & Cheek, 2022)	VAK 72<br>WT crude

		GT crude<br>WU(T) crude<br>WU(K) crude

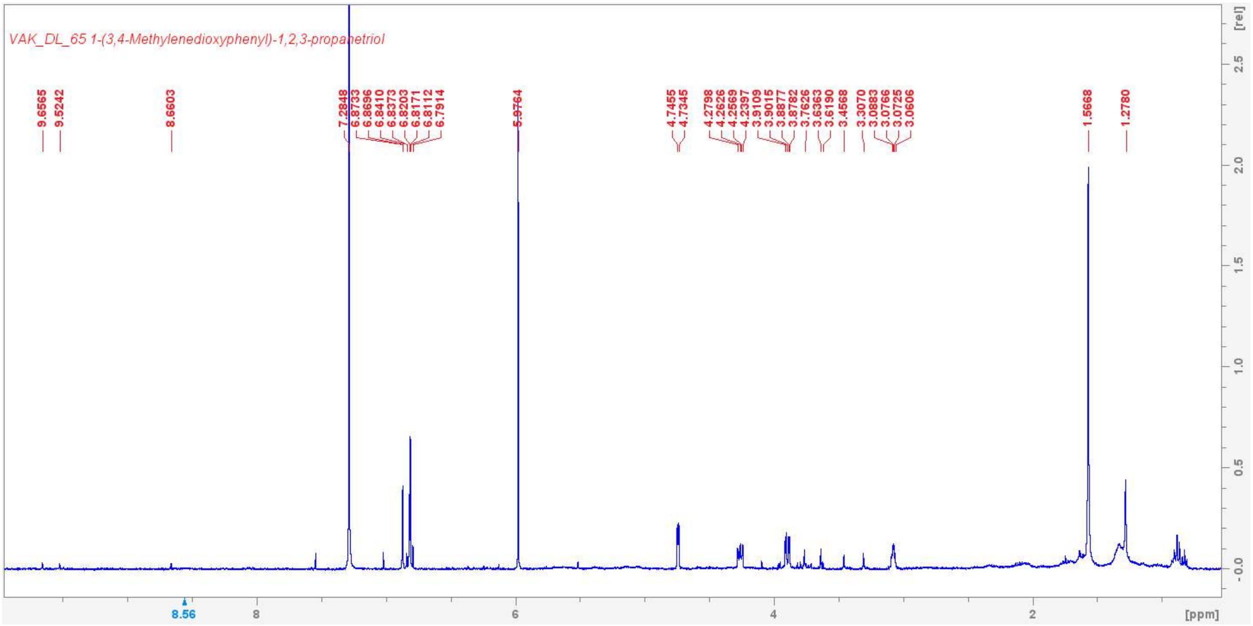

H^1 NMR spectra of the lignan, 1-(3,4-Methylenedioxyphenyl)-1,2,3-propanetriol

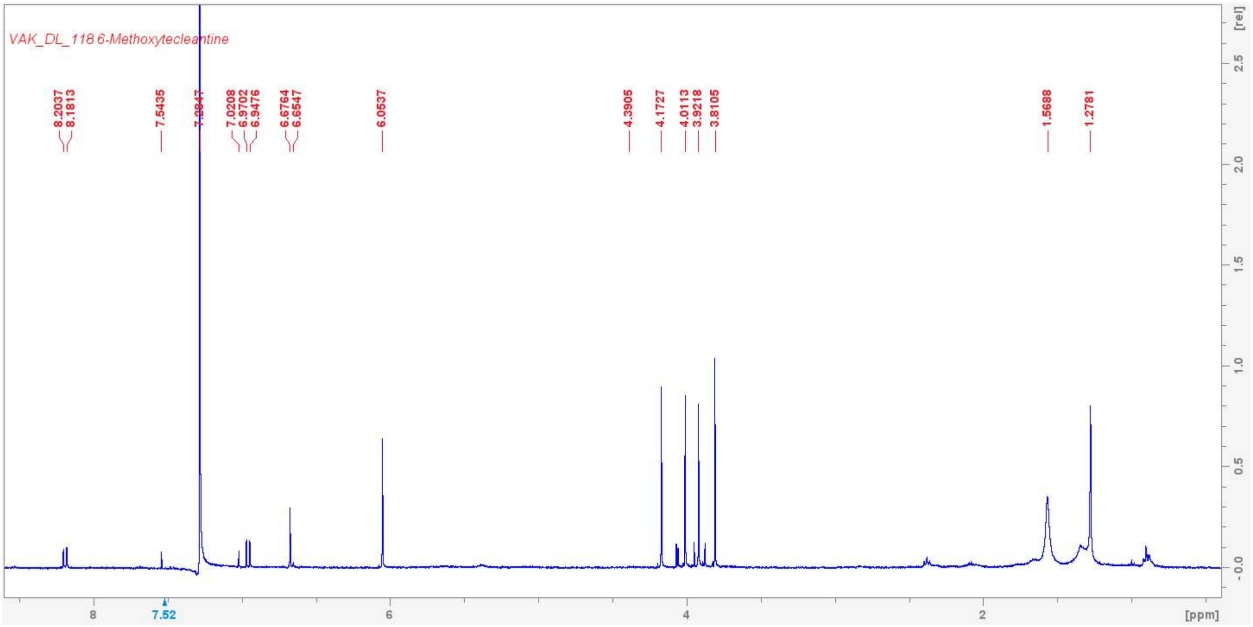

H^1 NMR spectra of 6-methoxytecleanthine

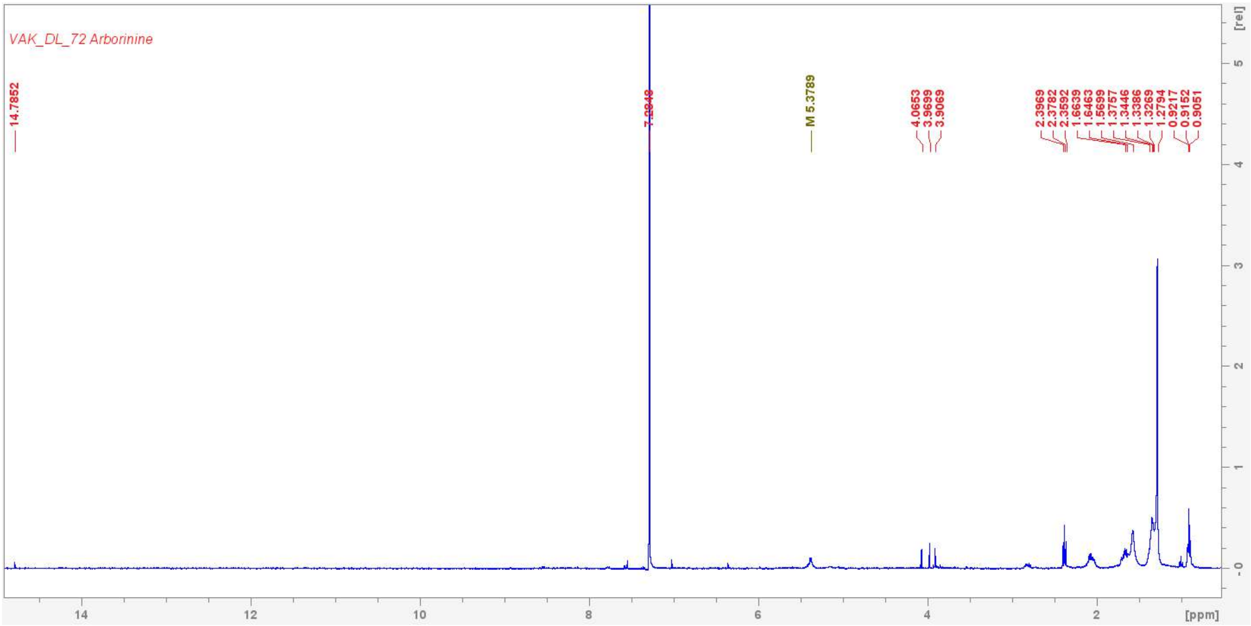

H[1] NMR spectra of arborinine

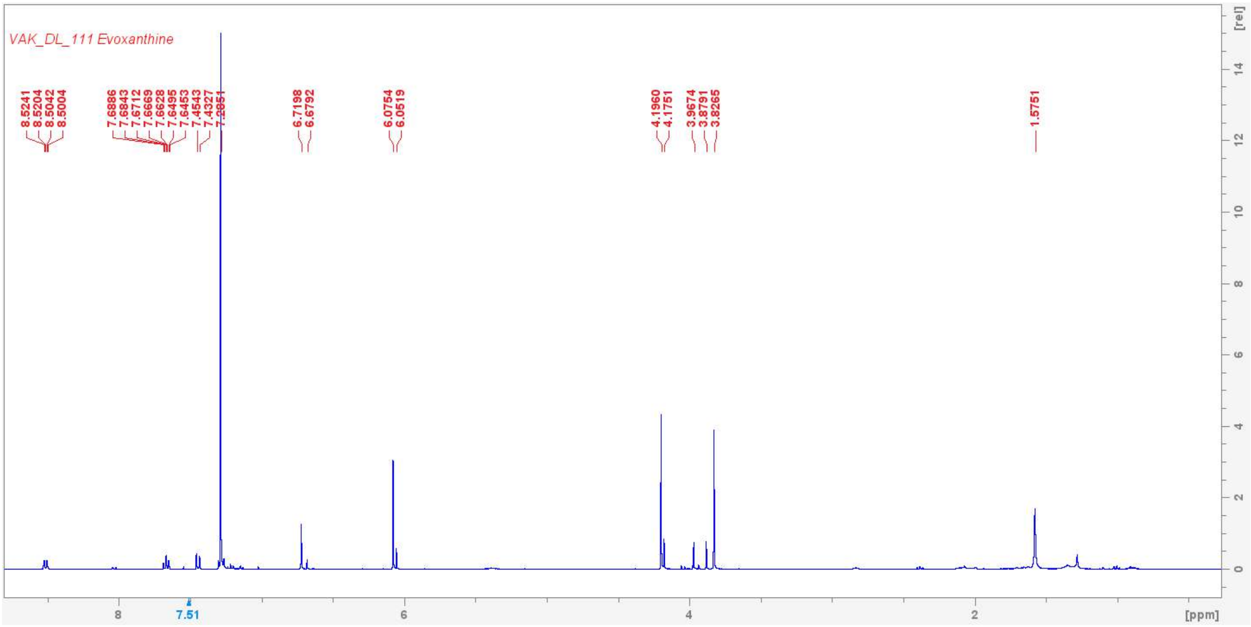

H[1] NMR spectra of evoxanthine

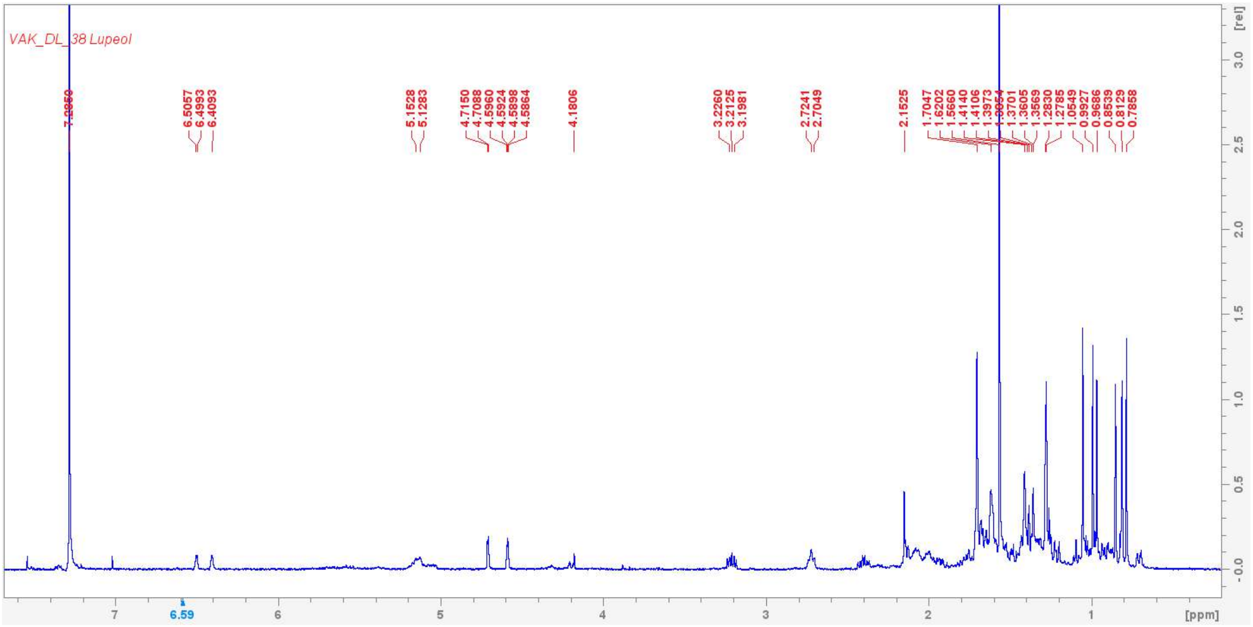

Figure 9. H^1 NMR spectra of lupeol

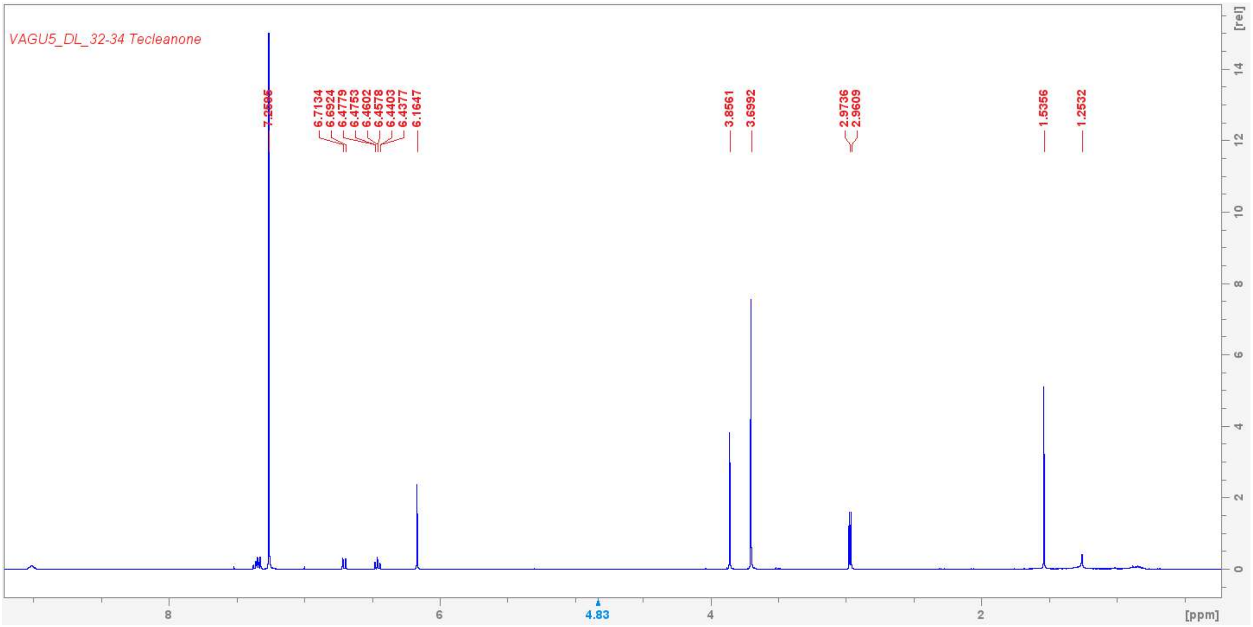

H^1 NMR spectra of tecleanone

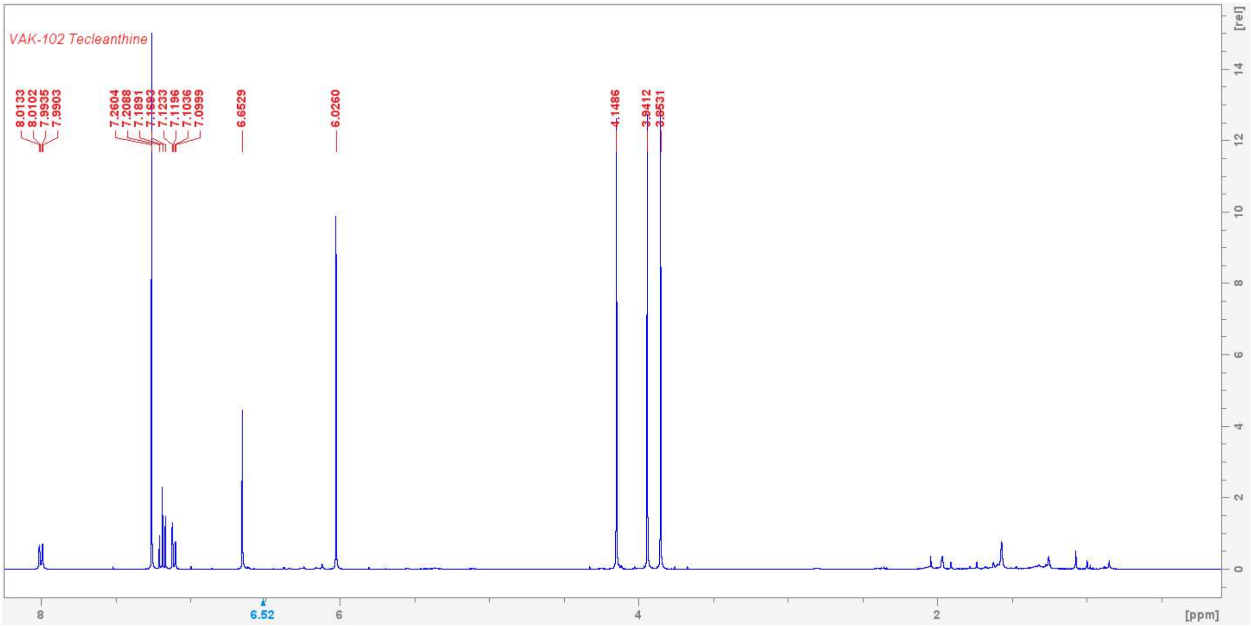

H^1 NMR spectra of tecleanthine
