# Supplementary figures and images for: Vepris amaniensis: a morphological, biochemical, and molecular investigation of a species complex
Source: PeerJ. 2024 Sep 25;12:e17881. doi: 10.7717/peerj.17881 (PMC11438429; doi:10.7717/peerj.17881)

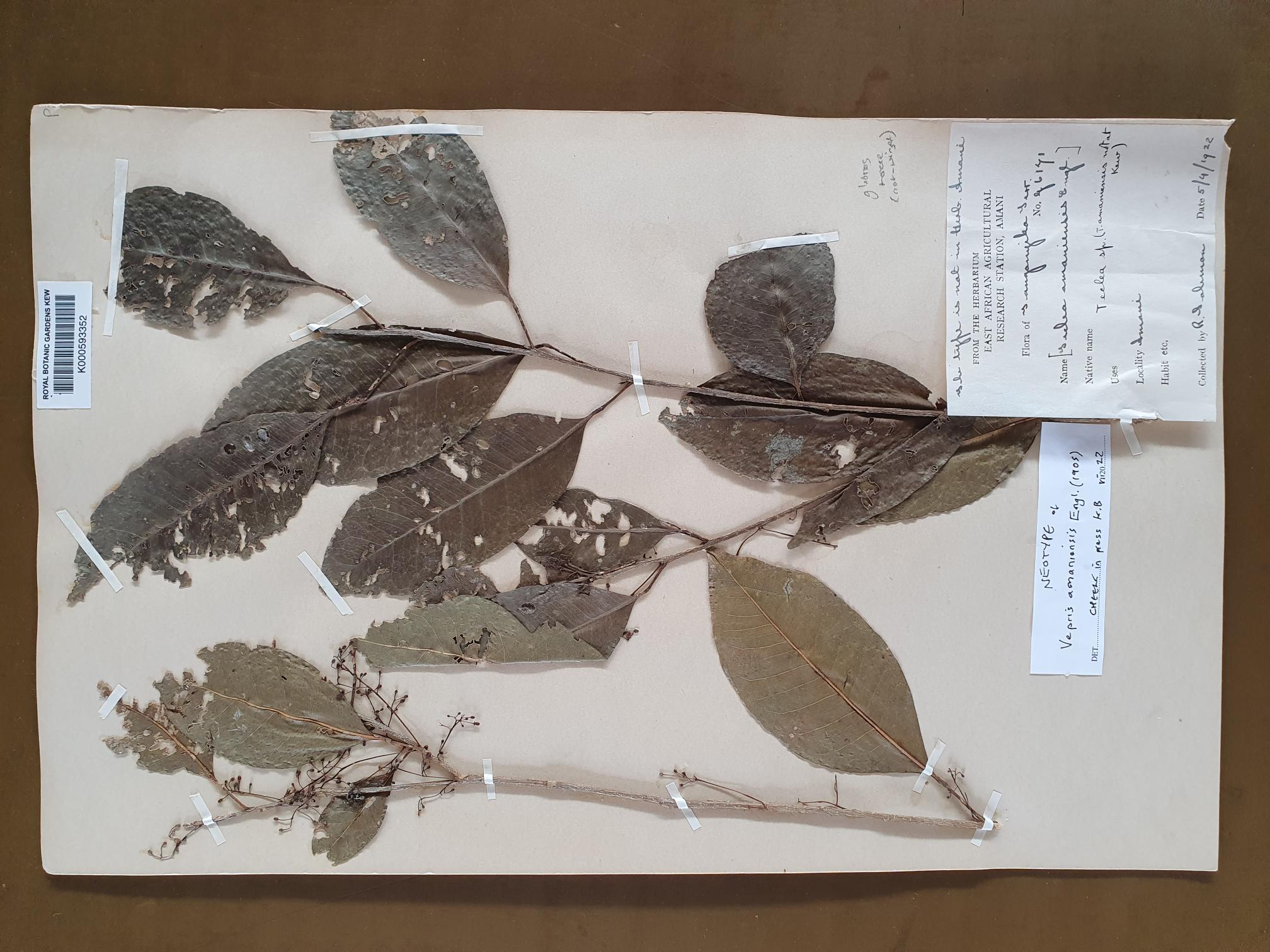

Supplement: Supplemental Information 7 — Image of neotype published by Cheek 2023 for Vepris amaniensis, Borhidi et al. 85340. [file peerj-12-17881-s007.jpg]
